# Association of Perceived Role Misidentification With Use of Role Identity Badges Among Resident Physicians

**DOI:** 10.1001/jamanetworkopen.2022.24236

**Published:** 2022-07-28

**Authors:** Michael B. Foote, Nina Jain, Benjamin N. Rome, Ersilia M. DeFilippis, Camille E. Powe, Maria A. Yialamas

**Affiliations:** 1 Department of Medicine, Memorial Sloan Kettering Cancer Center, New York, New York; 2 Department of Medicine, Brigham and Women’s Hospital, Boston, Massachusetts; 3 Division of Pharmacoepidemiology and Pharmacoeconomics, Department of Medicine, Brigham and Women’s Hospital and Harvard Medical School, Boston, Massachusetts; 4 New York Presbyterian-Columbia University Irving Medical Center, New York; 5 Diabetes Unit, Division of Endocrinology, Massachusetts General Hospital, Boston

## Abstract

**Importance:**

Role misidentification of resident physicians occurs frequently and is associated with decreased well-being.

**Objective:**

To evaluate the role misidentification and burnout rates among resident physicians after disbursement of role identity badges.

**Design, Setting, and Participants:**

This quality improvement study was conducted during the 2018 to 2019 academic year. Residents in 13 surgical and nonsurgical residency programs at 2 large academic medical centers (Massachusetts General Hospital and Brigham and Women’s Hospital) were eligible to receive the intervention and complete 2 surveys (before and after the intervention). Data were analyzed from December 4, 2021, to February 7, 2022.

**Intervention:**

Role identity badges that displayed “Doctor” and could be attached to mandatory hospital identification badges were distributed to residents in August 2018 at Massachusetts General Hospital and in March 2019 at Brigham and Women’s Hospital. Residents were not required to wear the badge.

**Main Outcomes and Measures:**

The primary outcome was self-reported role misidentification at least once per week during the previous 3 months. The change from pre– to post–badge distribution surveys was assessed with McNemar's test. A secondary outcome was any reduction in the frequency of role misidentification after badge distribution. Multivariable logistic regression was used to assess the association between reduced frequency of role misidentification and demographic characteristics. A separate analysis evaluated the change in self-reported burnout after badge distribution.

**Results:**

A total of 161 residents (39%) completed both surveys, which included 79 men (49%), 72 (45%) who were younger than 30 years, 20 (12%) with an underrepresented in medicine status, and 74 (46%) who were in surgical specialties. The proportion of residents reporting at least weekly role misidentification decreased from 50% (n = 81 of 161) before badge distribution to 35% (n = 57 of 161; *P* < .001) after badge distribution. Female residents were more likely to report reduced role misidentification frequency after receiving a badge compared with male residents (adjusted odds ratio, 2.32; 95% CI, 1.18-4.63; *P* = .01). Residents who wore badges demonstrated no change in burnout before vs after badge distribution (39% [n = 33 of 85] vs 34% [29 of 85]; *P* = .87) compared with an increase among residents who did not wear a badge (27% [n = 15 of 55] vs 45% [n = 25 of 55]; *P* = .03).

**Conclusions and Relevance:**

This study found that the distribution of role identity badges was associated with less frequent perception of role misidentification among resident physicians across specialties, particularly among female residents. Role identity badges were a well-received, low-cost intervention that could be used to reduce role misidentification and address burnout among residents.

## Key Points

**Question:**

Is the distribution of role identity badges (displaying “Doctor”) to resident physicians associated with reduced perception of role misidentification and burnout?

**Findings:**

In this quality improvement study of 161 residents at 2 academic medical centers, the proportion of residents who reported at least weekly role misidentification decreased after badge distribution, and the proportion reporting burnout increased in residents who did not wear a badge and did not change in those who wore a badge.

**Meaning:**

Findings of this study suggest that role identity badges are a low-cost intervention to reduce role misidentification and address burnout, particularly among female residents.

## Teaser

This quality improvement study assesses the implications of providing medical trainees with a wearable badge that indicates their physician role on the patient care team.

## Introduction

Hospitalized patients often cannot identify their inpatient physician among the assembly of heterogeneous professionals fulfilling different care roles.^1^ When patients and families experience this uncertainty, their questions can be misdirected to the wrong clinician, and patient care may suffer.^1,2^ Role misidentification, or the incorrect identification of a clinician’s contribution to the care team, may also adversely affect the well-being of resident physicians. Frequent role misidentification may contribute to imposter syndrome and burnout among clinicians.^3,4,5^ Residents are particularly susceptible to role misidentification and its adverse consequences.^3,5^

Our previous cross-sectional survey of more than 200 residents across multiple specialties found that role misidentification was a pervasive problem.^5^ Female residents were more likely to report role misidentification compared with male residents, and role misidentification was more common among surgical than medical residents.^5^ Frequent role misidentification was associated with burnout.^5^ Similar results have been replicated at other academic medical centers.^6,7,8^

Given the crisis of increasing burnout among residents, an intervention targeting role misidentification offers an opportunity to improve resident well-being. In a pilot study, role identity badges displaying “Doctor” were distributed to internal medicine residents; badge disbursement was found to be associated with reduced rates of role misidentification and improved day-to-day resident work experience, particularly among female residents.^9^ However, the association between role misidentification and burnout after badge distribution has not been completely characterized, particularly across multiple specialties.

In this study, we expanded the “doctor” role identity badge intervention to 13 additional residency programs at 2 large academic medical centers. We aimed to evaluate the role misidentification and burnout rates among resident physicians after disbursement of the role identity badges.

## Methods

This quality improvement study was approved by the Mass General Brigham Institutional Review Board. All participants submitted informed consent forms to be included in this voluntary study. We followed the Standards for Quality Improvement Reporting Excellence (SQUIRE) reporting guideline.

### Setting and Participants

We performed a prospective, longitudinal evaluation of role misidentification and burnout before and after the distribution of role identity badges in the 2018 to 2019 academic year. We included all residents in residency programs that provided clinical rotations at Brigham and Women’s Hospital except those in programs that had distributed similar badges before this study began (internal medicine, general surgery, emergency medicine, and obstetrics/gynecology). We also included general surgery, radiology, and neurosurgery residents at Massachusetts General Hospital.

### Intervention

New role identity badges were distributed to the residents at Massachusetts General Hospital in August 2018 and to all residents at Brigham and Women’s Hospital in March 2019; the Brigham and Women’s Hospital badges were delayed by 6 months for logistical reasons. The badges displayed “Doctor” and could be attached to mandatory hospital identification badges.^9^ Badges were disbursed by program representatives, advertised by email, and available for pickup at centralized locations; residents were not required to wear a role identity badge.

### Outcomes and Survey Design

The primary study outcome was frequent role misidentification, defined as self-reported misidentification at least once per week within the previous 3 months, based on our previous study.^5^ A secondary outcome was any reduction in frequency of role misidentification between the pre–badge and post–badge distribution surveys.

A pre–badge distribution survey was disseminated to all eligible residents in July 2018. These residents were sent a post–badge distribution survey 3 months after badge distribution. Surveys asked residents to report their demographic information, perceived frequency of role misidentification, and symptoms of burnout (eTable 1 in the Supplement). Residents were classified as underrepresented in medicine if they self-identified their race and ethnicity as biracial or multiracial, Black or African American, Hispanic, Native American or American Indian, or Native Hawaiian or Other Pacific Islander. Burnout was self-assessed using a validated single-item scale with this prompt: “Overall, based on your definition of burnout, how would you rate your level of burnout” (eTable 1 in the Supplement).^10^ This approach was chosen to minimize survey fatigue and maximize response rate. The post–badge distribution survey also asked about resident acquisition and use of the badge and perceived implications of the badge for their day-to-day experience.

We selected representative favorable and constructive comments about the role identity badge intervention to identify the implications for future similar interventions. Both pre–badge and post–badge distribution surveys were designed by our research team based on experience from the pilot study.^9^ The surveys were administered and managed using the REDCap electronic data capture tool (Vanderbilt University), which allowed branching logic and anonymous linking of the pre–badge and post–badge distribution surveys.^11^ Participating residents received multiple reminder emails and a $10 gift certificate per completed survey.

### Statistical Analysis

We used the McNemar test for paired analysis of the change in the proportion of residents reporting role misidentification at least once per week before and after the badges were distributed. We similarly measured the change in self-reported burnout,^10^ which was dichotomized from a 5-point scale into burnout (scores of ≥3 points) or no burnout (scores of 1 or 2 points). The primary intention-to-treat analysis included all residents who completed both the pre–badge and post–badge distribution surveys regardless of whether they received or wore a badge or whether they reported receiving a badge from a different source before the study began.

For the secondary outcome, we measured the self-reported decrease in role misidentification frequency between the 2 surveys, defined as at least a 1-point reduction on a 7-point ordinal scale ranging from “never” to “more than once per day” (eTable 1 in the Supplement). We used multivariable logistic regression to assess the association between improvement in self-reported role misidentification frequency and demographic characteristics, including sex, age (<30 vs ≥30 years), underrepresented in medicine status, postgraduate year (1-2 vs ≥3), and program type (surgical vs nonsurgical^12^).

To address potential temporal confounding, we performed sensitivity analyses to compare changes between the pre–badge and post–badge distribution surveys in self-reported role misidentification and burnout among residents who received and wore a role identity badge vs 2 internal control groups: (1) residents who did not receive or wear a role identity badge and (2) residents in a program without a formal badge distribution but who received a badge from another source before this study.

All statistical analyses were performed using R, version 4.0.3 (R Foundation for Statistical Computing). All tests were 2-sided, and *P* < .05 indicated significance. Data were analyzed from December 4, 2021, to February 7, 2022.

## Results

**Figure 1.**
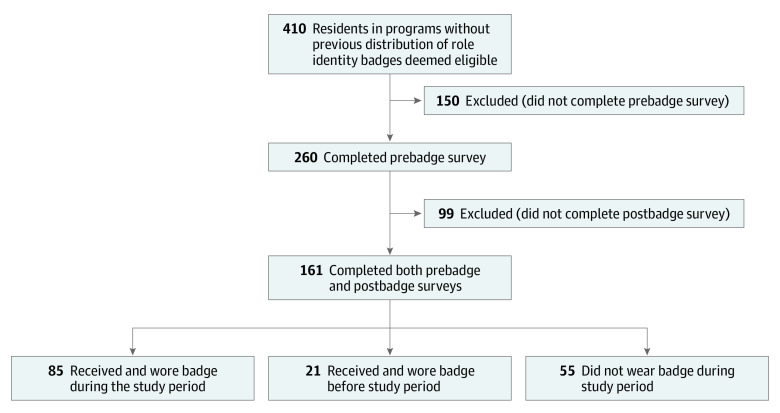
Flowchart of Participants

Of the 410 eligible resident physicians, 260 residents (63%) completed the pre–badge distribution survey; the 161 residents (39%) who completed both the pre–badge and post–badge distribution surveys represented the main study population (Figure 1). Among the 161 residents, 78 (48%) were women and 79 (49%) were men, 74 (46%) were in surgical specialties, 72 (45%) were younger than 30 years, and 20 (12%) had an underrepresented in medicine status (Table 1; eTable 2 in the Supplement). Residents who completed both surveys had characteristics similar to the 99 residents who completed only the pre–badge distribution survey (eTable 3 in the Supplement). In the pre–badge distribution survey, 81 residents (50%) reported frequent role misidentification. Female residents were more likely to report frequent role misidentification in the pre–badge distribution survey than male residents (79% [n = 62 of 78] vs 24% [n = 19 of 79]).

**Table 1.** Characteristics of Residents Stratified by Self-reported Role Misidentification Before Badge Disbursement

Characteristic	Residents, No. (%)		
	Misidentified (n = 81)^**a**^	Not misidentified (n = 80)	All residents (n = 161)
Age, y			
≥30	39 (44)	50 (56)	89 (55)
<30	42 (58)	30 (42)	72 (45)
Sex			
Female	62 (79)	16 (21)	78 (48)
Male	19 (24)	60 (76)	79 (49)
Unspecified	0	4 (100)	4 (2)
Race and ethnicity^b^			
Non-URM status	68 (52)	63 (48)	131 (81)
URM status	12 (60)	8 (40)	20 (12)
Not specified	1 (10)	9 (90)	10 (6)
PGY			
1-2	34 (59)	24 (41)	58 (36)
≥3	47 (46)	56 (54)	103 (64)
Program			
Nonsurgical	42 (48)	45 (52)	87 (54)
Surgical	39 (53)	35 (47)	74 (46)

Abbreviations: PGY, postgraduate year; URM, underrepresented in medicine.

^a^ Defined as at least weekly role misidentification within the previous 3 months.

^b^ Race and ethnicity were self-reported. Residents who self-identified their race and ethnicity as biracial or multiracial, Black or African American, Hispanic, Native American or American Indian, or Native Hawaiian or Other Pacific Islander were categorized under URM status.

Of the 161 residents who completed both surveys, 85 (53%) received and wore a role identity badge during the study period, 55 (34%) did not receive a badge or received a badge but did not wear it, and 21 (13%) wore a badge that was acquired from another source before this study. Among the 106 residents who wore the badge during the study, including residents who acquired a badge before (n = 21) or after (n = 85) the pre–badge distribution survey, 65 (61%) were women. In contrast, most residents who did not wear the badge were men (73% [n = 40 of 55]). Role identity badge distribution was estimated to be feasible for the different residency programs through email advertising and announcements at group residency events.

In the main study population, the proportion of residents who reported frequent role misidentification decreased from 50% (n = 81 of 161) in the pre–badge distribution survey to 35% (n = 57 of 161; *P* < .001) in the post–badge distribution survey. Of the 81 residents who reported initial frequent role misidentification, 35 (43%) did not report frequent role misidentification in the post–badge distribution survey. Eleven residents (14%) who did not initially report frequent role misidentification subsequently reported frequent role misidentification after the badges were distributed.

**Figure 2.**
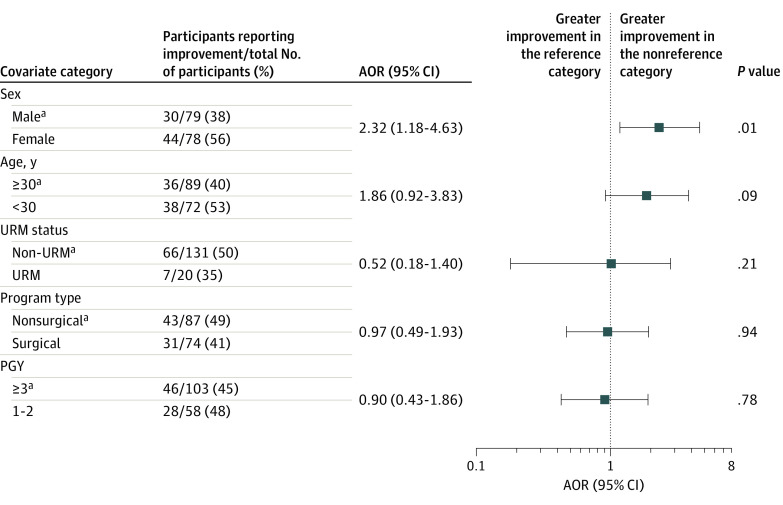
Association Between Study Characteristics and Improvement in Role Misidentification Improvement was defined as reduction of at least 1 ordinal level of role misidentification in the post–badge vs pre–badge distribution survey. Adjusted odds ratios (AORs) were adjusted for all other variables. Residents who did not identify sex or race and ethnicity categorized as underrepresented in medicine (URM) status (n = 10 of 161) were excluded. PGY indicates postgraduate year. ^a^Reference group for each dichotomous covariate pair.

Seventy-four residents (46%) reported any degree of improvement in role misidentification after badge distribution compared with before badge distribution (eFigure in the Supplement). In a multivariable analysis, female residents were more likely to report reduced role misidentification frequency compared with male residents (adjusted odds ratio, 2.32; 95% CI, 1.18-4.63; *P* = .01) (Figure 2). Other demographic characteristics were not associated with changes in role misidentification.

**Figure 3.**
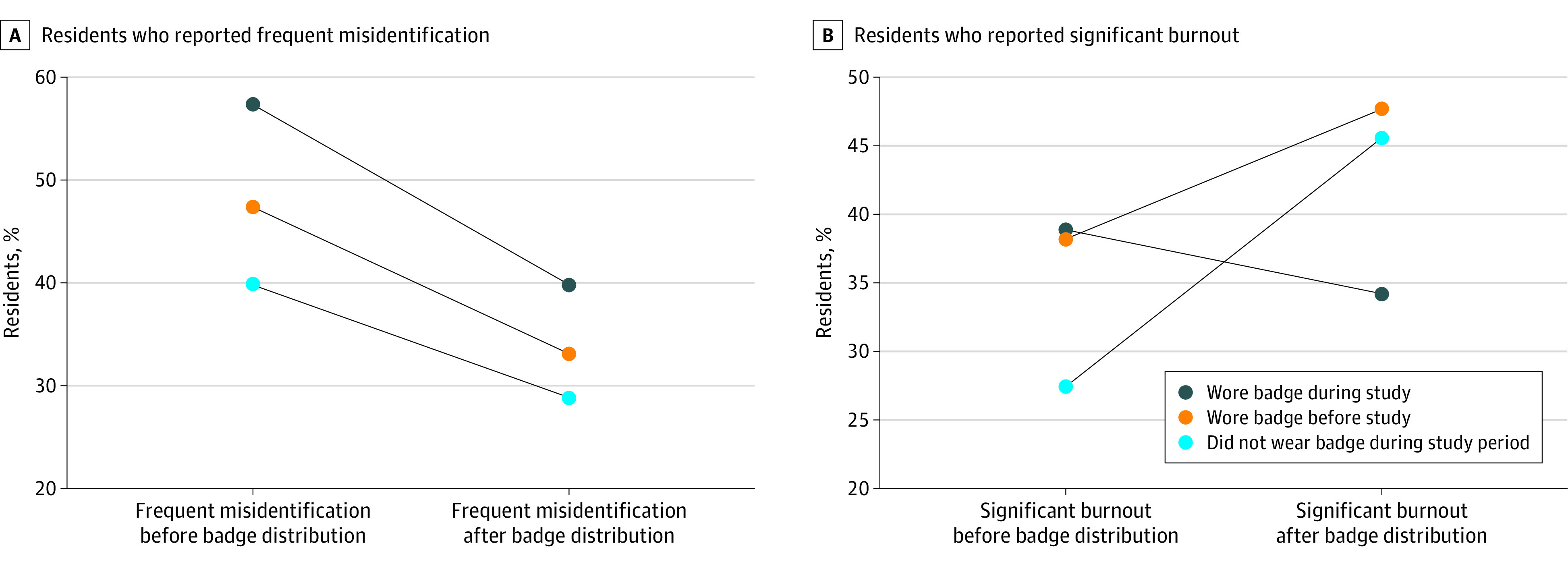
Change in Percentage of Residents Who Reported Frequent Role Misidentification and Significant Burnout Before and After Badge Distribution

In a sensitivity analysis of the 85 residents who received and wore a badge during the study period, the percentage of residents who reported frequent role misidentification decreased from 58% (n = 49 of 85) to 40% (n = 34 of 85; *P* = .001) during the study (Figure 3A). In comparison, residents who did not receive or wear a badge had a smaller decrease in frequent role misidentification from 40% (n = 22 of 55) to 29% (n = 16 of 55; *P* = .11). Cohort characteristics for each subgroup are provided in eTable 4 in the Supplement.

Overall, 51 residents (35%) reported symptoms of burnout in the pre–badge distribution survey compared with 64 residents (40%; *P* = .32) in the post–badge distribution survey. In a separate sensitivity analysis of 85 residents who received and wore a badge during the study period, the percentage of residents who reported symptoms of burnout did not significantly change from 39% (n = 33 of 85) to 34% (n = 29 of 85; *P* = .87) (Figure 3B). In contrast, the proportion of residents who reported symptoms of burnout increased among those who did not receive or wear a badge from 27% (n = 15 of 55) in the pre–badge distribution survey to 45% (n = 25 of 55; *P* = .03) in the post–badge distribution survey (Figure 3B). Burnout also increased among the 21 residents who wore a badge they received before the pre–badge distribution survey.

Among the 106 residents who received and wore the badge at any time (including before study initiation), 61% (n = 65) reported that the badge “positively affects my day-to-day experience,” including 72% (n = 50) of the 69 female residents. The free-text comments by residents suggested that wearing the badge was associated with improved confidence, improved patient-physician relationship, reduced patient request for nonphysician tasks, and encouraging conversations about gender-based bias in medicine (Table 2). Constructive feedback from residents included the observation that a role identity badge may be noticed more by the staff than patients, concerns about the bulkiness of the badge, and suggestions to expand badge distribution to all team members.

**Table 2.** Select Feedback About the Role Identity Badge Intervention

Favorable feedback	Implications
“It allows patients and family members to quickly identify [me] as a physician, which gets rid of awkward mistakes and clarifications and allows for faster building of rapport.”	By preventing role misidentification, role identity badges can improve the physician-patient relationship.
“It gives me more confidence that the assumption of whether I am the doctor is no longer there. Gives me the ability to just be the physician and concentrate on doing my job, instead of worrying about any assumptions of my role.”	Role identity badges can increase confidence and reduce the stereotypical imposter syndrome in physicians.
“I have had patients thank me for wearing the ‘doctor’ identification badge so they knew my role in their clinic visit. I feel I am asked to do nonphysician tasks less, such as helping change linens or boost patients while I am doing my own tasks, because my role is clearer.”	Role identity badges can clarify roles for both patients and other members of the care team.
“Overall, the most important impact has been as a conversation piece. Many people, including patients, colleagues, and friends have noticed and commented on the badge, which has led to a conversation about why they are necessary and the continued problem of sexism in medicine.”	Role identity badges can initiate a conversation about gender-based discrimination in medicine.
**Constructive feedback**	**Potential solutions**
“I think the hospital staff who spend all day looking [at] these badges have become familiar with the colors/names on them and it makes them less likely to misidentify me. I'm not sure the patients really look at them ... so that hasn't really changed.”	Role identity badges may not be sufficient to improve patient identification of care team members.
“Some nonphysician clinicians may view these as a way to reinforce the traditional medical hierarchy.… A hospital-wide initiative for role-labeled badges may be warranted to foster mutual respect for each others' roles and improve interdisciplinary communication.”	Role identity badges should be distributed to all team members (not just physicians) to avoid competition and promote collaboration.
“I don't wear it because it makes my ID significantly larger and more cumbersome to wear.”	Clear role description could be included on mandatory hospital identification badges to avoid the need for 2 separate badges.

## Discussion

In this prospective study of resident physicians at academic medical centers, we found an association between the distribution of the role identity badge and reduced perception of role misidentification, particularly among female residents who were twice as likely to report less frequent role misidentification after receiving the badge than male residents. As found in a previous study,^5^ perceived role misidentification was common among female residents in this study. Nearly 4 of 5 female residents reported role misidentification in the pre–badge distribution survey compared with 1 in 4 male residents (79% vs 24%). The role identity badge intervention was well received across multiple residency programs and residents; most residents across specialties wore the optional badges and reported beneficial changes to their day-to-day life. These results support the expanded use of role identity badges as low-cost tools for reducing role misidentification and potentially improving the wellbeing of clinicians with a high prevalence of role misidentification, including female residents.^5,9^

The results confirm and expand the findings of a previous pilot study, in which role identity badges were beneficial and well received among internal medicine residents in a single training program.^9^ In addition, the results are aligned with findings from a similar study at Mayo Clinic, which reported that role misidentification by patients, nonphysician team members, and other physicians decreased significantly after the distribution of a role identity badge to residents in multiple specialties.^6^ Moreover, multiple studies have documented that female residents have higher rates of reported role misidentification than male residents and experience more pronounced reductions in the frequency of role misidentification after the distribution of role identity badges.^6,7,8,9^

The proportion of residents who reported role misidentification decreased over the course of the study, including among residents who did not receive or wear a badge. This finding could be explained by improvements in resident confidence over the academic year or may be associated with selection bias given that 38% of residents who completed the pre–badge distribution survey did not complete the post–badge distribution survey. In addition, pre–badge distribution rates of reported role misidentification were lower among residents who did not receive and/or wear a badge, suggesting that badges may have been used by residents who were most likely to benefit from this intervention. Despite the higher prevalence of role misidentification among surgical residents,^5^ we did not find that surgical residents had greater or reduced improvement in role misidentification compared with nonsurgical residents. Uniform requirements for surgical specialties are unique; scrub-based attire is shared among different medical professionals and may be a factor in greater role misidentification. Moreover, role identity badges may not have been visibly worn in sterile operating room environments. Additional measures may be warranted to address role misidentification among surgical trainees.

This study was conducted over the course of an academic year, during which burnout might be expected to worsen. In a subcohort of residents who did not receive or wear a badge, a significant increase in burnout over the year was observed (from 27% to 45%). This increase was not found in residents who received the badge during the study period, suggesting that improved role misidentification may have attenuated the usual increase in burnout. Burnout rates were also slightly higher in a small group of residents who received a badge before the study period (n = 21). This group reported a lower initial prevalence of role misidentification before badge distribution; given their previous badge use, burnout in this group may have been less associated with role misidentification.

Critiques of the role identity badge intervention included resident concerns that the badges were viewed “as a way to reinforce the traditional medical hierarchy.” Appropriate recognition of all care team members is important to both patients’ understanding of their care and productive interdisciplinary communication. In addition, accurate recognition of occupational roles is important to the well-being of all staff, not just the physicians. Although this study focused on resident physicians, Brigham and Women’s Hospital has since distributed similar role identity badges to all patient-facing staff members.

### Limitations

This study has several limitations. A relatively small number of residents at 2 academic medical centers were included, which could limit generalizability. In addition, some residency programs were excluded because they had distributed similar badges before this study. Although we had a greater than 50% response rate to each survey, only 38% of residents completed both surveys; it is possible that nonrespondents had experiences that were different from those of the respondents. The demographic characteristics were similar across all residents at the 2 academic medical centers. Role misidentification was self-reported and therefore subject to recall bias or response bias because residents were not blinded to the purpose of the study. Burnout was assessed with a single-item scale. This abbreviated scale has been found to be associated with validated inventories,^10^ and using a single-item approach reduced the time required to complete the survey.

The study was nonrandomized. Although residents who did not receive or wear a badge served as a comparative internal control group, it is possible that the decreased role misidentification over time was confounded by temporal patterns. We are not aware of other concurrent interventions that addressed role misidentification during the time frame of this study. Future investigation is needed to continue the characterization of the long-term implications of role misidentification for the well-being of residents, including those from communities that are traditionally underrepresented in medicine.

## Conclusions

Distribution of role identity badges was associated with less frequent perception of role misidentification, particularly among female resident physicians. The role identity badge intervention was favorably received by most residents and represents a low-cost and scalable tool in reducing role misidentification and addressing burnout among residents.

## Notes

Supplement. **eTable 1.** Study Pre-Badge and Post-Badge Survey Instrument **eTable 2.** Detailed Characteristics of 161 Resident Participants in the Study Cohort Stratified by Self-Reported Misidentification Before Badge Disbursement **eTable 3.** Characteristics of Residents Evaluated for Study Eligibility Based on Taking Both the Pre-Badge and Post-Badge Survey Versus Only the Pre-Badge Survey **eTable 4.** Detailed Characteristics of 161 Resident Participants in the Study Cohort Stratified by Timing and Use of the “Doctor” Badge **eFigure.** Change in Resident-Reported Misidentification Ordinal Scores in the Pre-Badge Survey and Post-Badge Survey
