# Supplementary material for: Association of Perceived Role Misidentification With Use of Role Identity Badges Among Resident Physicians
Source: JAMA Netw Open. 2022 Jul 28;5(7):e2224236. doi: 10.1001/jamanetworkopen.2022.24236 (PMC9335144; doi:10.1001/jamanetworkopen.2022.24236)
Supplement: Supplement. — eTable 1. Study Pre-Badge and Post-Badge Survey Instrument eTable 2. Detailed Characteristics of 161 Resident Participants in the Study Cohort Stratified by Self-Reported Misidentification Before Badge Disbursement eTable 3. Characteristics of Residents Evaluated for Study Eligibility Based on Taking Both the Pre-Badge and Post-Badge Survey Versus Only the Pre-Badge Survey eTable 4. Detailed Characteristics of 161 Resident Participants in the Study Cohort Stratified by Timing and Use of the “Doctor” Badge eFigure. Change in Resident-Reported Misidentification Ordinal Scores in the Pre-Badge Survey and Post-Badge Survey [file jamanetwopen-e2224236-s001.pdf]

Supplemental Online Content

Foote MB, Jain N, Rome BN, DeFilippis EM, Powe CE, Yialamas MA. Association of perceived role misidentification with use of role identity badges among resident physicians. *JAMA Netw Open*. 2022;5(7):e2224236.
doi:10.1001/jamanetworkopen.2022.24236

eTable 1. Study Pre-Badge and Post-Badge Survey Instrument

eTable 2. Detailed Characteristics of 161 Resident Participants in the Study Cohort Stratified by Self-Reported Misidentification Before Badge Disbursement

eTable 3. Characteristics of Residents Evaluated for Study Eligibility Based on Taking Both the Pre-Badge and Post-Badge Survey Versus Only the Pre-Badge Survey

eTable 4. Detailed Characteristics of 161 Resident Participants in the Study Cohort Stratified by Timing and Use of the "Doctor" Badge

eFigure. Change in Resident-Reported Misidentification Ordinal Scores in the Pre-Badge Survey and Post-Badge Survey

This supplemental material has been provided by the authors to give readers additional information about their work.

© 2022 Foote MB et al. *JAMA Network Open.*

eTable 1. Study Pre-Badge and Post-Badge Survey Instrument

**Study pre-badge survey instrument**
In the last three months spent rotating in a Partners hospital, how often have you personally been misidentified as a different member of the care team other than a physician?<br><br>More than once per day<br>About once per day<br>A few times per week<br>About once per week<br>About once per month<br>Less than once per month<br>Never
IF above question is not answered "Never"<br>In the last three months spent rotating in a Partners hospital, which of the following individuals has misidentified you as a member of the care team other than a doctor? (Select all that apply)<br><br>Patients<br>Patients' families<br>Nurses<br>Physician Assistants or Nurse Practitioners<br>Clinical Support Staff (e.g. nurse assistants, nutritionists, physical therapists, medical assistants, social workers)<br>Non-clinical support staff (e.g. food services, environmental services)<br>Residents or fellows (including those in and not in your program)<br>Attending Physicians
IF above question is not answered "Never"<br>In the last three months spent rotating in a Partners hospital, which of the following individuals was the MOST likely to misidentify you as a member of the care team other than a doctor?<br><br>Patients<br>Patients' families<br>Nurses<br>Physician Assistants or Nurse Practitioners<br>Clinical Support Staff (e.g. nurse assistants, nutritionists, physical therapists, medical assistants, social workers)<br>Non-clinical support staff (e.g. food services, environmental services)<br>Residents or fellows (including those in and not in your program)<br>Attending Physicians
IF above question is not answered "Never"<br>Do you feel that being misidentified as a member of the care team other than a doctor negatively affects your overall well-being in the hospital?

© 2022 Foote MB et al. *JAMA Network Open.*

Yes<br>No
If yes, please explain.<br><br>[Free Text Response]
Overall, based on your definition of burnout, how would you rate your level of burnout?<br><br>1 - I enjoy my work. I have no symptoms of burnout<br>2- Occasionally I am under stress, and I don't always have as much energy as I once did, but I don't feel burned out<br>3- I am definitely burning out and have one or more symptoms of burnout, such as physical and emotional exhaustion<br>4- The symptoms of burnout that I'm experiencing won't go away. I think about frustration at work a lot<br>5- I feel completely burned out and often wonder if I can go on. I am at the point where I may need some changes or may need to seek some sort of help
What is your current level of training? (If you are on research, what is the highest PGY that you have completed?)<br><br>PGY1<br>PGY2<br>PGY3<br>PGY4<br>PGY5<br>PGY6<br>PGY7<br>PGY8+
What is your residency program?<br><br>Anesthesiology<br>Dermatology (including Medicine/Dermatology)<br>Emergency Medicine<br>Internal Medicine<br>Internal Medicine / Pediatrics<br>Medical Genetics<br>Neurology<br>Neurosurgery<br>OB/GYN<br>Oral Medicine<br>Orthopedic surgery<br>Otolaryngology / ENT<br>Pathology<br>Pediatrics

© 2022 Foote MB et al. *JAMA Network Open.*

Plastic Surgery<br>Psychiatry<br>Radiology<br>Radiation Oncology<br>Surgery<br>Urology
What is your residency program's affiliation?<br><br>Brigham and Women's Hospital<br>Massachusetts General Hospital<br>Combination program (split equally between both campuses)
What is your age?<br><br>Less than 25 years old<br>25 – 29 years old<br>30 – 34 years old<br>35 – 39 years old<br>40 years old or greater
What is your gender identity?<br><br>Male<br>Female<br>Other [With free text response]<br>I prefer not to disclose my gender
What is your ethnicity?<br><br>Hispanic<br>Non-Hispanic<br>Prefer not to disclose
What is your race?<br><br>White<br>Black or African American<br>Native American or American Indian<br>Asian<br>Native Hawaiian or Other Pacific Islander<br>Biracial or multiracial<br>Prefer not to disclose
Would you be willing to be contacted further by a researcher about the experiences you report here?<br><br>Yes<br>No

© 2022 Foote MB et al. *JAMA Network Open.*

**Study post-badge survey instrument**
In the past three months, how often have you personally been misidentified as a different member of the care team other than a physician while at work?<br><br>More than once per day<br>About once per day<br>A few times per week<br>About once per week<br>About once per month<br>Less than once per month<br>Never
IF above question is not answered "Never"<br>In the last three months spent rotating in a Partners hospital, which of the following individuals has misidentified you as a member of the care team other than a doctor? (Select all that apply)<br><br>Patients<br>Patients' families<br>Nurses<br>Physician Assistants or Nurse Practitioners<br>Clinical Support Staff (e.g. nurse assistants, nutritionists, physical therapists, medical assistants, social workers)<br>Non-clinical support staff (e.g. food services, environmental services)<br>Residents or fellows (including those in and not in your program)<br>Attending Physicians
IF above question is not answered "Never"<br>In the last three months spent rotating in a Partners hospital, which of the following individuals was the MOST likely to misidentify you as a member of the care team other than a doctor?<br><br>Patients<br>Patients' families<br>Nurses<br>Physician Assistants or Nurse Practitioners<br>Clinical Support Staff (e.g. nurse assistants, nutritionists, physical therapists, medical assistants, social workers)<br>Non-clinical support staff (e.g. food services, environmental services)<br>Residents or fellows (including those in and not in your program)<br>Attending Physicians
Overall, based on your definition of burnout, how would you rate your level of burnout?<br><br>1 - I enjoy my work. I have no symptoms of burnout<br>2- Occasionally I am under stress, and I don't always have as much energy as I once did, but I don't feel burned out

© 2022 Foote MB et al. *JAMA Network Open.*

3- I am definitely burning out and have one or more symptoms of burnout, such as physical and emotional exhaustion<br>4- The symptoms of burnout that I'm experiencing won't go away. I think about frustration at work a lot<br>5- I feel completely burned out and often wonder if I can go on. I am at the point where I may need some changes or may need to seek some sort of help
Have you ever received a separate badge to attach to your identification badge that clearly identifies you as a "Doctor" or "Physician" in large text?<br><br>I received a badge and wear it<br>I received a badge but do not wear it<br>I have never receive a badge
IF above question answered" I received and wear it" or "I received a badge but do not wear it"<br>When did you receive this "Doctor" or "Physician" identification badge?<br><br>Within the past 6 months<br>More than 6 months ago
IF above question answered" I received a badge but do not wear it"<br>What is the reason you do not wear the "Doctor" badge? (check all that apply)<br><br>I do not like the appearance of the badge<br>I do not feel like I need the badge<br>I feel that wearing the badge would have negative consequences<br>Other [Free text response]
IF above question answered "I received a badge and wear it"<br>Since wearing your doctor badge, are you more or less likely to be misidentified as a member of the care team other than a physician?<br><br>Less likely to be misidentified<br>No change<br>More likely to be misidentified
IF above question answered "I received a badge and wear it"<br>Since wearing your doctor badge, are you more or less likely to be misidentified by the following individuals<br>*(GRID, options include: Less likely to be misidentified, no change, More likely to be misidentified, N/A)*<br><br>Patients<br>Patients' families<br>Nurses<br>Physician Assistants or Nurse Practitioners<br>Clinical Support Staff (e.g. nurse assistants, nutritionists, physical therapists, medical assistants, social workers)

© 2022 Foote MB et al. *JAMA Network Open.*

Non-clinical support staff (e.g. food services, environmental services)<br>Residents or fellows (including those in and not in your program)<br>Attending Physicians
IF above question answered "I received a badge and wear it"<br>Overall, how does wearing a "Doctor" badge affect your day-to-day experience in the hospital?<br><br>Positively affects my day-to-day experience<br>No effect on my day-to-day experience<br>Negatively affects my day-to-day experience<br>No opinion
IF above question answered "Positively affects my day-to-day experience" or "Negatively affects my day-to-day experience"<br>Please explain how the "Doctor" badge affects your day-to-day experience in the hospital.<br><br>[Optional free text response]
Do you have any additional positive or negative feedback about "Doctor" identification badges?<br><br>[Free text response]
What is your current PGY Year? (If you are on research, what is the highest PGY that you have completed?)<br><br>PGY1<br>PGY2<br>PGY3<br>PGY4<br>PGY5<br>PGY6<br>PGY7<br>PGY8+
What is your residency program<br><br>Anesthesiology<br>Dermatology (including Medicine/Dermatology)<br>Emergency Medicine<br>Internal Medicine<br>Medical Genetics<br>Neurology<br>Neurosurgery<br>OB/GYN<br>Oral Medicine<br>Orthopedic surgery<br>Otolaryngology / ENT

© 2022 Foote MB et al. *JAMA Network Open.*

Pathology<br>Plastic Surgery<br>Psychiatry<br>Radiology<br>Radiation Oncology<br>Surgery<br>Urology
What is your gender identity?<br><br>Male<br>Female<br>Other [With free text response]<br>I prefer not to disclose my gender
What is your ethnicity?<br><br>Hispanic<br>Non-Hispanic<br>Prefer not to disclose
What is your race?<br><br>White<br>Black or African American<br>Native American or American Indian<br>Asian<br>Native Hawaiian or Other Pacific Islander<br>Biracial or multiracial<br>Prefer not to disclose
Would you be willing to be contacted further by a researcher about the experiences you report here?<br><br>Yes<br>No

© 2022 Foote MB et al. *JAMA Network Open.*

eTable 2. Detailed Characteristics of 161 Resident Participants in the Study Cohort Stratified by Self-Reported Misidentification Before Badge Disbursement

Characteristic	Misidentified Pre-Badge (n=81)	Not Misidentified Pre-Badge (n=80)	All Subjects (N=161)
Age-no. (% by row)			
25-29	42 (58%)	30 (42%)	72 (45%)
30-34	35 (45%)	43 (55%)	78 (48%)
35-39	4 (40%)	6 (60%)	10 (6%)
Over 40	0 (0%)	1 (100%)	1 (1%)
Gender-no. (% by row)			
Female	62 (79%)	16 (21%)	78 (48%)
Male	19 (24%)	60 (76%)	79 (49%)
Not Specified	0 (0%)	4 (100%)	4 (2%)
Race-no. (% by row)			
Black/African American	4 (100%)	0 (0%)	4 (2%)
Asian	18 (49%)	19 (51%)	37 (23%)
White	53 (52%)	48 (48%)	101 (63%)
Native American or American Indian	0 (0%)	1 (100%)	1 (1%)
Multiracial	5 (62%)	3 (38%)	8 (5%)
Not Specified	1 (10%)	9 (90%)	10 (6%)
Ethnicity-no. (% by row)			
Hispanic	5 (42%)	7 (58%)	12 (7%)
Non-Hispanic	72 (53%)	64 (47%)	136 (84%)
Not Specified	4 (31%)	9 (69%)	13 (8%)
PGY-no. (% by row)			
1	14 (54%)	12 (46%)	26 (16%)
2	20 (62%)	12 (38%)	32 (20%)
3	29 (49%)	30 (51%)	59 (37%)
4	11 (38%)	18 (62%)	29 (18%)
5	5 (56%)	4 (44%)	9 (6%)
6	1 (33%)	2 (67%)	3 (2%)
7	1 (50%)	1 (50%)	2 (1%)
8	0 (0%)	1 (100%)	1 (1%)
Program-no. (% by row)			
Dermatology	9 (69%)	4 (31%)	13 (8%)
Neurology	7 (26%)	20 (74%)	27 (17%)
Neurosurgery	3 (60%)	2 (40%)	5 (3%)
Oral Medicine	3 (60%)	2 (40%)	5 (3%)

© 2022 Foote MB et al. *JAMA Network Open.*

Orthopedic Surgery	5 (45%)	6 (55%)	11 (7%)
Otolaryngology	6 (86%)	1 (14%)	7 (4%)
Pathology	3 (33%)	6 (67%)	9 (6%)
Plastic Surgery	1 (33%)	2 (67%)	3 (2%)
Psychiatry	15 (65%)	8 (35%)	23 (14%)
Radiation Oncology	5 (56%)	4 (44%)	9 (6%)
Radiology	0 (0%)	1 (100%)	1 (1%)
Surgery	23 (56%)	18 (44%)	41 (25%)
Urology	1 (14%)	6 (86%)	7 (4%)

© 2022 Foote MB et al. *JAMA Network Open.*

eTable 3. Characteristics of Residents Evaluated for Study Eligibility Based on Taking Both the Pre-Badge and Post-Badge Survey Versus Only the Pre-Badge Survey

Characteristic	Pre-badge survey only (n = 99)	Pre-badge and Post-badge survey (n = 161)	Combined Group (N=260)
Age-no. (% by column)			
30 and Older	59 (60%)	89 (55%)	148 (57%)
Under 30	40 (40%)	72 (45%)	112 (43%)
Gender-no. (% by column)	6 (60%)	10 (100%)	4 (40%)
Female	44 (44%)	78 (48%)	122 (47%)
Male	53 (54%)	79 (49%)	132 (51%)
Unspecified	2 (2%)	4 (3%)	6 (2%)
Race-no. (% by column)[1]			
Non-URM	82 (83%)	131 (81%)	213 (82%)
URM	12 (12%)	20 (12%)	32 (12%)
Not Specified	5 (5%)	10 (6%)	15 (6%)
PGY-no. (% by column)			
PGY 1-2	35 (35%)	58 (36%)	93 (36%)
PGY 3 or More	64 (65%)	103 (64%)	167 (64%)
Program-no. (% by column)			
Non Surgical Program	45 (45%)	87 (54%)	132 (51%)
Surgical Program	54 (55%)	74 (46%)	128 (49%)

[1]Residents that self-identified as a race or ethnicity as African American, Hispanic, Native American, Native Hawaiian or other Pacific Islander, or Biracial/Multiracial were categorized as under-represented in medicine (URM)

© 2022 Foote MB et al. *JAMA Network Open.*

eTable 4. Detailed Characteristics of 161 Resident Participants in the Study Cohort Stratified by Timing and Use of the "Doctor" Badge

Characteristic	Received and Wore Badge Within Trial Period (n=85)	Wore Badge But Received Before Trial Period (n=21)	Did Not Wear Badge Within Trial Period (n=55)	All Subjects (N=161)
Age-no. (% by column)				
25-29	38 (45%)	12 (57%)	22 (40%)	72 (45%)
30-34	39 (46%)	9 (43%)	30 (55%)	78 (48%)
35-39	8 (9%)	0 (0%)	2 (4%)	10 (6%)
Over 40	0 (0%)	0 (0%)	1 (1.8%)	1 (1%)
Gender-no. (% by column)				
Female	51 (60%)	14 (67%)	13 (24%)	78 (48%)
Male	33 (39%)	6 (29%)	40 (73%)	79 (49%)
Not Specified	1 (1%)	1 (5%)	2 (4%)	4 (2%)
Race-no. (% by column)				
Black/African American	2 (2.4%)	1 (5%)	1 (2%)	4 (2%)
Asian	21 (25%)	6 (29%)	10 (18%)	37 (23%)
White	56 (66%)	12 (57%)	33 (60%)	101 (63%)
Multiracial	4 (5%)	0 (0%)	4 (7%)	8 (5%)
Native American or American Indian	0 (0%)	0 (0%)	1 (2%)	1 (1%)
Not Specified	2 (2%)	2 (10%)	6 (11%)	10 (6%)
Ethnicity-no. (% by column)				
Hispanic	6 (7%)	3 (14%)	3 (5%)	12 (7%)
Non-Hispanic	78 (92%)	15 (71%)	43 (78%)	136 (84%)
Not Specified	1 (1%)	3 (14%)	9 (16%)	13 (8%)
PGY-no. (% by column)				
1	16 (19%)	4 (19%)	6 (11%)	26 (16%)
2	16 (19%)	7 (33%)	9 (16%)	32 (20%)
3	34 (40%)	6 (29%)	19 (35%)	59 (37%)
4	13 (15%)	4 (19%)	12 (22%)	29 (18%)
5	4 (5%)	0 (0%)	5 (9%)	9 (6%)
6	2 (2%)	0 (0%)	1 (2%)	3 (2%)
7	0 (0%)	0 (0%)	2 (4%)	2 (1%)
8	0 (0%)	0 (0%)	1 (2%)	1 (1%)
Program-no. (% by column)				
Dermatology	5 (6%)	2 (10%)	6 (11%)	13 (8%)
Neurology	15 (18%)	4 (19%)	8 (15%)	27 (17%)
Neurosurgery	1 (1%)	1 (5%)	3 (5%)	5 (3%)
Oral Medicine	4 (5%)	1 (5%)	0 (0%)	5 (3%)

© 2022 Foote MB et al. *JAMA Network Open.*

Orthopedic Surgery	2 (2%)	0 (0%)	9 (16%)	11 (7%)
Otolaryngology	0 (0%)	2 (10%)	5 (9%)	7 (4%)
Pathology	5 (6%)	1 (5%)	3 (5%)	9 (6%)
Plastic Surgery	1 (1%)	0 (0%)	2 (4%)	3 (2%)
Psychiatry	16 (19%)	4 (19%)	3 (5%)	23 (14%)
Radiation Oncology	5 (6%)	1 (5%)	3 (5%)	9 (6%)
Radiology	1 (1%)	0 (0%)	0 (0%)	1 (1%)
Surgery	27 (32%)	3 (14%)	11 (20%)	41 (25%)
Urology	3 (4%)	2 (10%)	2 (4%)	7 (4%)

© 2022 Foote MB et al. *JAMA Network Open.*

eFigure. Change in Resident-Reported Misidentification Ordinal Scores in the Pre-Badge Survey and Post-Badge Survey

Pre-Badge Survey \ Post-Badge Survey	Never	Less than once per month	Once per month	Once per week	A few times per week	Once per day	More than once per day	Total
Never	68%	18%	5%	5%	0%	0%	3%	38
Less than once per month	61%	17%	11%	6%	6%	0%	0%	18
Once per month	29%	25%	21%	21%	4%	0%	0%	24
Once per week	27%	10%	17%	37%	10%	0%	0%	30
A few times per week	17%	9%	22%	0%	35%	9%	9%	23
Once per day	7%	0%	27%	7%	40%	20%	0%	15
More than once per day	8%	0%	15%	0%	38%	23%	15%	13
Total	58	21	25	20	24	8	5	161

Each cell compares individual residents' reported frequency of misidentification on the pre-badge (row) and post-badge (column) surveys.

© 2022 Foote MB et al. *JAMA Network Open.*
